# Development and Implementation of a Hybrid Wireless Sensor Network of Low Power and Long Range for Urban Environments

**DOI:** 10.3390/s21020567

**Published:** 2021-01-14

**Authors:** Juan Bravo-Arrabal, J. J. Fernandez-Lozano, Javier Serón, Jose Antonio Gomez-Ruiz, Alfonso García-Cerezo

**Affiliations:** Robotics and Mechatronics Lab, Andalucía Tech, Universidad de Málaga, 29071 Málaga, Spain; jfl@uma.es (J.J.F.-L.); jseron@uma.es (J.S.); janto@uma.es (J.A.G.-R.); ajgarcia@uma.es (A.G.-C.)

**Keywords:** LoRa, hybrid wireless sensor network, mobile sensors, urban environment monitoring

## Abstract

The urban population, worldwide, is growing exponentially and with it the demand for information on pollution levels, vehicle traffic, or available parking, giving rise to citizens connected to their environment. This article presents an experimental long range (LoRa) and low power consumption network, with a combination of static and mobile wireless sensors (hybrid architecture) to tune and validate concentrator placement, to obtain a large coverage in an urban environment. A mobile node has been used, carrying a gateway and various sensors. The Activation By Personalization (ABP) mode has been used, justified for urban applications requiring multicasting. This allows to compare the coverage of each static gateway, being able to make practical decisions about its location. With this methodology, it has been possible to provide service to the city of Malaga, through a single concentrator node. The information acquired is synchronized in an external database, to monitor the data in real time, being able to geolocate the dataframes through web mapping services. This work presents the development and implementation of a hybrid wireless sensor network of long range and low power, configured and tuned to achieve efficient performance in a mid-size city, and tested in experiments in a real urban environment.

## 1. Introduction

Currently, 5G technology is being researched and implemented to extract its maximum potential, in terms of high transfer rates, high bandwidth and very low latency [1,2,3,4,5,6]. If 4G gave rise to the era of entertainment, allowing high-definition video to be viewed in streaming, 5G is going to represent a new paradigm that already calls into question the future usefulness of wired telecommunications [7,8]. However, it is still in an initial stage of deployment, where it relies on 4G infrastructure, although providing more throughput [9,10]. It is expected that along 2021, latency will be reduced to 1 ms [11,12], which will allow the development of applications focused on instantaneous responses, such as remote surgical operations [13], intelligent traffic control [14], monitoring of parking spaces available in a city [15,16,17], or full-scale conversations using holograms that are reproduced instantly, even from the mobile phone [18]. As 5G evolves, it is worth reflecting on how LPWAN (Low-Power Wide-Area Network) technologies will fit in and how they should be adapted to this imminent scenario, as well as weighing up the costs and benefits they will have within this new world of interconnected things and people [19,20,21,22,23,24,25]. At the moment, there is a competition in the LPWAN market, where Sigfox, NB-IoT and long range (LoRa) [26] intend to be the future standard of an ecosystem in which everything will literally be on the web, changing how the cities are conceived [27]. These technologies operate in the sub-gigahertz electromagnetic spectrum band, known as Industrial, Scientific and Medical (ISM), which is license-free and provides high noise robustness, achieving long signal ranges, but only for low data rates (several kbps).

Cities have become a niche for the IoT market, and society demands information about what is happening in urban but also in remote landscapes [28]. In fact, it is not only important to find consensus among companies to reach an LPWAN standard, but also to find social acceptance and above all, to get governments and society involved. Emerging countries, where a huge potential of smart cities is foreseen, are growing technologically in this field [29,30]. One of the problems to control and manage in smart cities is traffic congestion, which also means a worrying escalation of pollution [31]. In this context, in the last five years, several IoT applications have been developed, making use of the different LPWAN options. The goal is to mitigate the hostile characteristics of urban environments [32] in terms of interferences, range and energy independence [33,34,35,36,37,38]. In addition, LoRaWAN, which is a kind of low power wide area network, has been used to develop wireless positioning techniques [39], even without a GPS antenna [40,41]. The latest research points to the union of Machine Learning and IoT as the most powerful tool to address these problems, being also an opportunity for the development of new urban applications [42,43].

When deploying a sensor network in an urban environment, key aspects, such as good line-of-sight between transmitter and receiver, the required coverage according to the use case, or the lifetime of the end-devices, have to be taken into account [44]. In the last few years, several authors have shown different node-location strategies to achieve good coverage and interconnectivity. A powerful approach is to rely on models to predict the performance of the network according to some of its key parameters, like gateway placement [45]. Other approaches make use of vehicular ad hoc networks (VANETs), in which there is no central authority to manage the nodes [46,47]. These networks allow for the replacement of damaged nodes or easy changes to the configuration of the deployed network. Some authors have proposed VANETs for real-time acquisition and monitoring [48], making use of cloud computing [46] and high-resolution maps [49]. Some algorithms have been proposed, such as the latency and coverage optimized data collection scheme for smart cities in [50], to improve the efficiency data collection using data mules, but in that case, LoRaWAN was ruled out because they did not want to depend on long-range wireless transmission issues. Other authors [51,52] are including Unmanned Aerial Vehicles (UAVs) in this type of network to extend coverage, simulating different mobility models to predict performance, and emphasizing the importance of Quality of Service (QoS). However, among the several available LPWAN technologies, there are limited works dealing with experimental results that may help in the configuration of new applications. In particular, for the case of LoRaWAN, very few works address the deployment in an actual city or in a big urban extension [53], being this a relevant point given the differences between their real and model-based behavior [54]. Besides, Hybrid Wireless Sensor Networks (H-WSN), including at least one mobile node, have not received attention, despite their suitability for a number of applications, including urban monitoring, but also an emergency response, among others [55,56,57].

This paper presents the development and implementation of a H-WSN [58] for an urban environment. The proposed network includes a mobile node playing a double role: data transmitter and concentrator. This way, the mobile node allows the validation of the gateway placements across the city where the network has been deployed. It has a modular architecture, which allows the use of various sensors depending on the application to be developed, including parts per million (ppm) of certain gases, temperature, humidity, pressure or GPS location. The data packets are captured by several gateways, one local and some external, strategically located to cover the largest urban area possible. The nodes have been configured in Activation By Personalization (ABP) mode, for multicasting operation, in order to count and geolocate the packets perceived by each concentrator node, and thus be able to distinguish their area of coverage. In addition, all the gateways are synchronized with an external database hosted on a web server, linked to Google My Maps services for monitoring, in real time, the information from the sensors, allowing each measurement to be associated with its position.

The main contributions of this article are:

(a) Development and implementation of a hybrid wireless sensor network of low power and long range in an urban environment, including static and mobile nodes, with a modular architecture, taking into account the characteristics of the area of interest (and including real-time presentation of information); (b) configuration and tuning of the hybrid wireless network to achieve efficient performance in a mid-sized city, including the location of gateways in the urban area; (c) experiments to test and validate the performance of the network in an urban environment.

The article is structured as follows: after this introduction, Section 2 briefly describes the most relevant technical aspects of LoRa technology, which serves as a context for the justification of the adopted solution. In Section 3, the hybrid network of concentrator nodes is presented and the elements and characteristics of the system are detailed. In addition, the mobile node is shown, which acts as a transmitter and sink for urban information. Section 4 is dedicated to the experiments designed to validate the system. Finally, in Section 5, the conclusions reached are presented.

## 2. Lora Technology

### 2.1. Overview

LoRa is a LPWAN technology capable of transmitting small data packets with some regularity. The protagonists of the communication are the end-devices, formed by two parts: a radiofrequency module with a transmission antenna, and a microprocessor, able to process the data acquired by certain sensors. LoRa corresponds to the network layer of the Open System Interconnection (OSI) model, while LoRaWAN refers to the link-layer (MAC), which establishes the performance of the network according to its three classes of devices [22,59,60]. The most important differentiating feature between the classes is that each one chooses when a transmitting node can receive data from a server through a gateway:

- Class A: The transmitting node remains in silent mode, while listening to possible configuration signals (coming through a gateway). It has two short reception windows, i.e., two offset times, and a configurable data rate. Transmissions from the server, or downlink (DL), are only permitted when communications from end-devices to the LoRa Network Server (LNS) through any gateway, known as uplink (UL), are successful. The concentrator node can respond only in one of the two reception slots. This is the type of device consuming the least energy.
- Class B: They use synchronization periods, called beacons, issued by the gateway. Therefore, this class is similar to A but also opens extra reception windows at scheduled times for DL messages from the server. This class is intended for applications requiring higher download data traffic (higher DL than above).
- Class C: Devices are always in silent mode, except when a sensor detects an event that has to be transmitted, so it is the class of device with the highest energy consumption. This allows planning for more reception windows (apart from the two standard slots present in all the classes) without prioritizing the success of UL communications. A trade-off occurs: more data can be transferred per unit of time, but at the cost of more consumption. So, the use of this class can be associated with continuity and consumption.

All three classes of devices can coexist on the same network (with the physical layer remaining unchanged), and each device can switch from one class to another [26], thus changing the way it operates. However, there is no way to tell the gateway which class of device is sending the information since it depends on the application. Communication is always two-way but half-duplex, i.e., transmitters can send the values acquired by the sensors, but they can also receive data from servers through gateways, such as an acknowledgment of receipt (ACK), but never at the same time or through the same channel. The LoRaWAN protocol is designed for applications connected to the Internet that operate with the data emitted by the transmitters. Therefore, depending on the class of device (A, B or C), the LoRa Network Server will have more or less time (reception window width per period) to try to respond through a gateway. Thus, class A devices have a shorter time for reception, while class C devices have a longer one, and class B is in between. The longer the time for reception, the higher the energy consumption. End-devices used in the experiments (Section 4) have been configured as class A, because this operation mode supports the longest battery life.

The physical layer (LoRa) operates on the ISM band (in the case of Europe, 863–870 MHz), which implies freedom of use but the transmitter nodes have to be programmed according to some limitations for the users:

- Transmission power is limited to 25 mW (14 dBm) for UL messages, i.e., The consumption equivalent to one light-emitting diode (LED). For messages traveling from applications to end-devices (DL), transmission is allowed up to half a watt (27 dBm).
- Duty cycle depends on the channel used and for transmission time of a node, and its value is between 0.1 and 1.
- Maximum gain allowed for an antenna is +2.5 dBi.

The time a message remains on the air is the period of time that the transmitting node must be consuming electrical power. LoRa radio waves are low-frequency light, that is to say, they travel at 300 km/ms. Therefore, the receiver has the possibility to get several times the message emitted by a certain end-device. However, apart from the duty cycle, other factors play a role in the communication being successful [61]:

- The line of sight (LoS) between transmitting and receiving antennas: the waves propagate, with the information acquired by the sensors, through the free space (medium). The presence of obstacles in the path of the messages weakens the intensity of the carrier signal [26], which is why it is important to take into account the Fresnel zones (ellipsoid-shaped volume of revolution covering the distance between the antennas). The offset between the waves contained in this volume must always be less than 180${}^{\circ }$. The center of the Fresnel zone is located in the middle of the distance between the end-device and the concentrator node. The reflection of the waves on obstacles, such as the ground (always present) can cause some of them to lag behind others. Thus, the gateways will receive waves directly but also from reflections. For this reason, the antennas should be placed outdoors and at height (the more the better). In addition, the antennas must be kept in a vertical position, being convenient that they are omnidirectional. The strength of the received signal is reduced as a result of the waves arriving out of phase with respect to those arriving directly. The next equation is used to quantify the losses (in dB) through the free space [62,63].
  (1) $$L_{f_{s}}=32.45+20\left(log\left(D\right)+log\left(f\right)\right),$$
  where
  $L_{f_{s}}$: Free space path loss, which quantifies the attenuation (in dB) of radio energy between transmitter node and gateways.
  *D*: Distance between transmitting (end-device) and receiving (gateway) antennas, in kilometers.
  *f*: Frequency in MHz.
- The Spread Factor (SF) is a redundancy factor programmed in the transmitter node. LoRa is configurable, which allows establishing communication strategies. The SF parameter allows us to widen the frequency spectrum, to a greater or lesser extent, using a certain number of bits that encode a symbol. The higher the number of bits, the lower the transfer rate and the greater the probability of receiving the messages [64], since they are more immune to the interference of the band. Spread bits vary between 7 and 12, and the less they are used, the less power the transmitter consumes.
- The length of the message has a significant influence on the transmission distance, so it is necessary to decide what information is really useful before programming the dataframes. It should also be noted that LoRa uses an error detection algorithm, known as Cyclic Redundancy Check (CRC), adding control bits to ensure that the message is received correctly [65]. So the message can be limited only up to a point.
- The problem of channel overlapping inherent to LoRa may make that a packet emitted by a given transmitter does not reach its destination [66,67].

Finally, for an end-device to be able to participate in a LoRaWAN network, it has to be customized and activated deploying the session keys. Only then, it can communicate with the LoRa Network Server.

## 3. Description of the System Based on a Hybrid Network of Concentrator Nodes

### 3.1. Overview

The developed application aims to analyze the operability of LoRa technology in an urban environment, using a hybrid network of concentrator nodes, which means that there are some gateways situated in fixed positions but also there is one mobile concentrator. Furthermore, several wireless sensors (end-devices), of different types, have been mounted on the vehicle that will act as the only mobile node of the network. This mobile node works both acquiring information and as a mobile sink, since it embarks a gateway besides the wireless sensors. LoRa modules have different probes in order to measure various environmental magnitudes from the surroundings of the vehicle and send them to the LoRa Network Server through both the embarked gateway and the static gateways around the city. Some of the LoRa modules include a GPS antenna in order to geo-locate each emitted data packet (current position of the vehicle), so every message sent by the sensor nodes onboard the vehicle can be traced even if they are not received by any static gateway (due to interference problems or obstacles). This serves to ensure that the emitted frames can be associated with an updated geo-localization, since the vehicle position is obtained thanks to a GPS node onboard the car. The goal is to provide coverage to the sensor nodes along the city of Málaga, a mid-sized city (around 500,000 inhabitants) in the Mediterranean coast of Spain. In order to sweep the maximum possible extension of the urban area, several gateways have been strategically placed on the roofs of various buildings, after having studied the distances and possible obstacles along the way. Each of these gateway sites is characterized by being at a different height, in a more or less complex environment and, consequently, have very different visibility. In total, five gateways have been set up in the city, after obtaining the necessary permits (Engineering School, Faculty of Tourism, El Ejido Government Pavilion and the International Spanish Centre, located in the neighborhood of El Palo), as well as the one located inside the vehicle (mobile transmitter/concentrator node). Thus, four fixed gateways and the mobile one have been used. The main goal of the system is to test LoRa technology in an urban environment. In order to do so, this work is structured around two views:

- On the one hand, the system architecture is based on fixed and mobile gateways, so that the number of lost packets by the concentrator nodes external to the mobile node can be counted, thus analyzing the different possible configurations of LoRa, as a parameterizable technology. The gateway onboard the vehicle permits to obtain all the dataframes which could not be captured by the static ones. After that, it is possible to establish the zones in the city with better coverage. This way, it is intended to arrange the static gateways up around the city in a useful serving way.
- On the other hand, a previous work provided a working solution to the problem in the same city [68]. That work, based on Zigbee technology, is used as a baseline to compare results and to verify any improvements of using LoRa in this scenario. For this purpose, the vehicle has followed the same route that in [68].

With this approach, the influence of existing obstacles in a city like Málaga on the reception of LoRa messages of different sizes has been studied, using different transmission configurations (Spread Factor), having several static receivers located in different places, in addition to the gateway located in the mobile node. The activation mode of LoRa nodes will also play a key role in the performance of the system (Section 3.3).

### 3.2. System Architecture and Implementation

**Figure 1:**
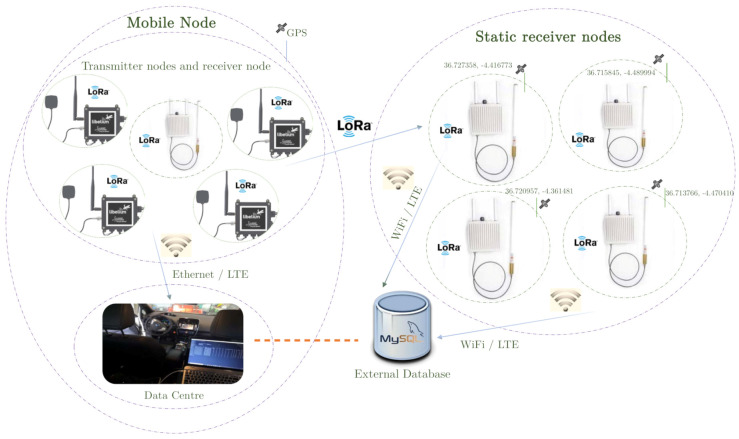
Hybrid gateway network-based system architecture.

The system includes two kinds of elements: transmitter nodes (remote sensors, also known as motes) and concentrator nodes (also known as gateways or packet forwarders). Actually, each gateway consists of a data hub node, i.e., a LoRa radio transceiver, and a Linux-based host, which has internal memory and allows connection to the Internet (via Ethernet or SIM card). At a higher level, the elements of the system are divided into two distinct groups (Figure 1):

- A mobile node, equipped with a gateway and several wireless sensor modules. This gateway, being at a short distance from the transmitting nodes, guarantees the reception of all transmitted packets, except those that collide because they use the same channel at the same moment. It is worth noting that in each sensor module there can be a single probe or more than one, so the same transmitter node can send more than one magnitude in its own dataframe, associated with its unique identifier.
- An external network of concentrator nodes (gateways) located in fixed locations in the city in order to cover the largest possible area, and thus be able to analyze the range of the LoRa packets in an urban environment.

The end-devices, onboard the vehicle, collect information from the urban environment, such as temperature, pressure, humidity, as well as the concentration of different gases such as CO, $CO_{2}$, NO, $NO_{2}$, $O_{3}$, and send it (UL) through the LoRa radio channels (half-duplex). In addition, each sensor module can be equipped with a GPS antenna, so that it is possible to associate the measurements taken by the sensors with the geo-location of the vehicle at a certain point in the journey. Latitude and longitude values are also sent via LoRa. Each node has been programmed to comply with the LoRa specifications (Section 2.1), so in order to avoid packet transmission failures that cause the current location to be lost, as well as to ensure a quick update of the vehicles’ position, a total of three GPS receivers were mounted in the vehicle.

The concentrator node located in the vehicle receives the information captured by the transmitter nodes and stores it, in real-time, in its internal memory, which allows us to save all the data even if the Internet is not available. In this way, the system can work offline. For that purpose, this gateway, and those in the static network outside the vehicle, host their own Message Queuing Telemetry Transport (MQTT) broker, which contains the topics to which MQTT clients can subscribe to obtain the information (published from end-devices) they desire. Thus, while the vehicle is driven by the route through the city, all the hosts (Linux-based) inside gateways run an MQTT client which subscribe to all the uplink packets that the packet forwarders receive via LoRa and, while receiving the information, store it in a plain text file in its own memory. It is important to notice that each of the gateways are set to generate a .txt file where the information taken by the wireless sensor network is saved. Each stored file will be processed locally by a program that decodes the frames, groups them according to their temporal occurrence, and dumps them into a common database outside the gateways, where the information captured by each end-device is organized in tables. The static concentrator nodes, located on different sites in the city, are also connected (via Ethernet or LTE) to the external database if they operate in the online mode. Otherwise (offline mode), data acquired by these gateways will be dump into the external database after the experiments. The mentioned operating modes (online and offline) are detailed in Section 3.4.

In this way, it is possible to treat the vehicle as a single mobile sensor node, which simultaneously acts as a mobile concentrator node. From small and individual messages collected by the nodes embarked in the vehicle, it is possible to build a large and common data packet related to the vehicle (seen as a whole), larger than the LoRa size limitation (222 bytes). The measures taken by all the gateways (including the one in the vehicle) are grouped in the external database. By means of software, all this information is managed and supervised.

In addition, each LoRa packet is associated with a location (latitude and longitude), so that it is possible to locate, in real-time, the occurrence of each measurement. From the database, queries can be made based on time ranges, managing to associate measurements that occurred in a small section of the route that were taken at a time prior to or after the time when the position of the vehicle was taken. In case of not having the position of a certain packet, the previous or the next one is associated to it, getting an insignificant error. This allows for traceability in the measures taken by the different sensors, but the really important thing is that it allows us to know, with certain accuracy, where the messages received by the static gateways were sent from, and where the packets were lost. Thanks to this system, it is possible to detect the areas of the city with less coverage, for a given configuration, thus being able to reorganize the static gateways to cover the largest possible area of the city.

The proposed system has been developed based on hardware components provided by Libelium (Zaragoza, Spain) and Multitech (Mounds View, MN, USA). The transmitter nodes have in common the Waspmote v12 module, and they include a LoRa communication module (Microchip, Chandler, AZ, USA) that provides the capability to use the LoRaWAN protocol. The concentrator nodes are based on the Conduit IP67 Base Station (Multitech MTCDTIP 220L), which is a ruggedized IoT gateway (Linux-based), which has been configured to be able to recognize the DevEUI of the transmitter nodes. It should be noted that these gateways do not have WiFi support, which is why an offline mode was initially developed (Section 3.4). However, if a specific gateway belongs to a Local Area Network (LAN), it can be accessed via SSH (port 22), using WiFi from a PC within that LAN.

**Figure 2:**
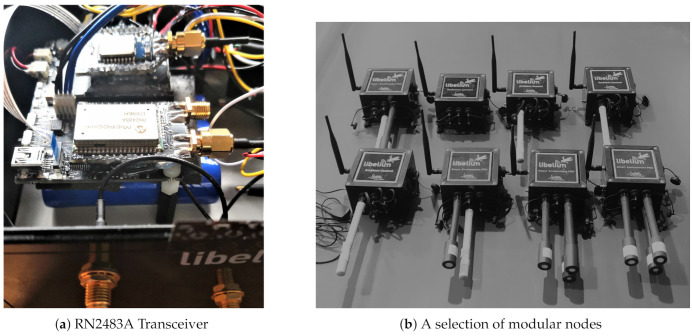
Long range (LoRa) end-devices.

The link between the mobile transmitting node and the static gateways, as well as with the one in the vehicle, has been implemented using the LoRaWAN protocol (868 MHz, Europe). It can transmit small information packets with a range, in theory, of several tens of kilometers (depending on visibility conditions and the established configuration, either of power, Spread Factor or data rates). The configuration of the network is quick and simple, allowing different configurations to be tried out in order to draw up strategies according to the type of city, with the possibility of adding more transmitter modules, both in the vehicle and in a static network. It is also easy to include other concentrator nodes in other areas of the city, which can cover a larger area, or provide redundancy in order to ensure the reception of all messages. In particular, the modules used were the following (Figure 2):

- Waspmote Plug Sense! Ambient Control LoRaWAN EU (GPS-ready). This module is capable of measuring the main environmental variables, admitting up to three types of probe: the 9370-P, capable of measuring temperature, pressure and humidity; the 9205-P and 9325-P, capable of measuring brightness values. The dynamics of these sensors are relatively fast, so they do not considerably influence the speed of transmission.
- Waspmote Plug Sense! Smart Environment PRO LoRaWAN EU (GPS-ready) This node allows to measure the concentration of various gases in the air of cities, such as $CO_{2}$, $NO_{2}$, CO and $O_{3}$, and can also measure temperature, pressure, humidity and luminosity.
- Waspmote Plug Sense! Radiation Control LoRaWAN EU (GPS-ready). It is capable of measuring radiation values using a Geiger sensor, but can also be used as a GPS module, configuring it to act as a GPS node, without measuring any other magnitude. This way, it emits frames of approximately 50 bytes with the latitude and longitude values of the mobile node, in ASCII.

**Figure 3:**
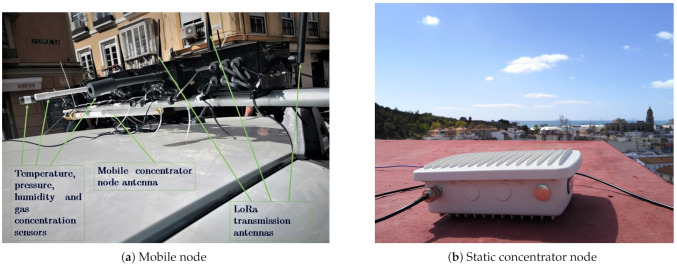
Elements of information transmission and reception.

Both end-devices and gateways are mounted in boxes with polycarbonate housing with IP65 protection (dust tight and water resistance enclosure), so that the system can be used outdoors. The Figure 3 shows the end-devices embarked in the vehicle and one of the static gateways used.

As mentioned above, all collected data are stored in the internal memory of the hub nodes, in the form of plain text files. Simultaneously, by means of software, all data are decoded, sorted and stored in the form of tables in an external database hosted on a web server. A MySQL database has been made to be the input for a graphical user interface (GUI), called lorApp, which is shown in the experiments section. This GUI has been created using QT Creator (a cross-platform C++ integrated development environment that simplifies GUI application development). It consists of a connection window and a setting window. The first one allows to download data received in any gateway of the network, and thus be able to dump its decoded content (from base64 to ASCII) into a database hosted on a local or web server (phpMyAdmin), depending on the operating mode (offline or online, see Section 3.4). Each gateway (the four static and the mobile one) has an MQTT broker running inside them, and with it, we can subscribe to all the information that goes through the server. This way, the number of packets stored by each of the gateways is known, as well as which gateways lost packets and how many. This is possible even if they are not connected to the Internet, due to the storage of the data in the internal memory of each concentrator node. The functions of the setting window are to:

- Indicate the concentrator node from which the data are extracted, using IP, user, password and port (SSH protocol).
- Access the database, indicating the IP, user, password, port and name of the database where the downloaded data are dumped.

This allows reconfiguring the parameters for future applications of the system, being able to establish better location strategies for the static concentrator nodes. The objective is to cover the largest possible area of the city with the least number of concentrator nodes. The number of packets received by the mobile node compared with the number received by each of the static gateways reveals the most efficient sites for locating them. The tables generated (each one associated with one end-device or specific node embarked in the vehicle) during the experiment have been imported, in Comma-Separated Values (CSV) format, into the Google MyMaps application, so that the data can be geo-located during or after the experiments. In addition, identifiers and colors have been established to find out which gateway received a particular packet broadcast from a specific location, as it is shown in the experiments.

### 3.3. Activation Modes

When the vehicle enters a specific area that is under the coverage of a static gateway, both the static and the mobile gateway must be able to receive the same packet transmitted by the same node at any given time in order to detect the white zones, that is to say, areas without coverage. Next, the two modes of LoRa node activation will be introduced, and the option adopted will be justified.

#### 3.3.1. Over the Air Activation

The Over The Air Activation (OTAA) mode is the most widely used mode because it provides greater security when connecting end-devices to the network server, since the network and application session keys (NwkSkey and AppSkey) are generated dynamically. These keys protect the integrity of the messages from the transmission to reception on the network server. Then, from the network server to the application server. Therefore, it is necessary to have both keys in order to decode a packet captured by a gateway, so the packets are protected against deciphering by gateways out of the system. Before activation, the LoRa Network Server must know and store three parameters:

- The Device Extended Unique Identifier (DevEUI), which identifies only the end-device (sensor node) and is similar to a MAC address, i.e., its fingerprint, which is registered in the device’s ROM. However, a DevEUI can always be assigned by modifying the value loaded by the operating system into the RAM, which is useful for privacy issues. This identifier consists of 64 bits.
- The Application Identifier (AppEUI), also 64 bits long, is analogous to an application port, through which the application server is accessed.
- The Application Key (Appkey) is a unique Advanced Encryption Standard (AES) 128-bit symmetric key, which must be stored in the network server and in the transmitter so that they can establish the join procedure. This secret key is known only by the end-device and the application with which it communicates, and it is used to determine the two session keys during the activation.

**Figure 4:**
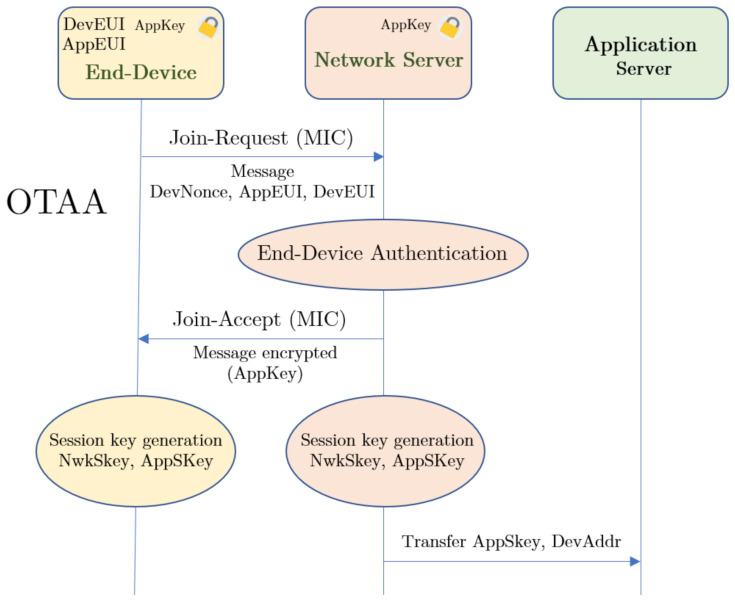
Over The Air Activation (OTAA) scheme.

The activation occurs in the air, and is renewed each time the device loses the connection, is turned off or rebooted, making it difficult for anyone to steal the session from the device due to the session keys also being renewed. As shown in Figure 4, the device requests to join the network server. To do this, it uses the pre-programmed DevEUI, AppEUI and Appkey, as well as a random number (DevNonce), which is used only once to avoid reply attacks [69,70].

Any gateway that can receive the request, forwards it to the network server, which will accept it if its AppKey matches the one the end-device has registered. Then, the Message Integrity Code (MIC) allows us to generate the session keys, which are unique per session to ensure the integrity of the information communicated. Only the gateway with the best Received Signal Strength Indicator (RSSI) will issue the response to the node.

In order to identify the application server, the AppEUI is used. This identifier is like a port number associated with an application, so transmitters will send data through a certain AppEUI when they are linked to the same application server. Since session keys are only generated when required (node reconnected) they cannot be compromised before joining. If the node changes its network, it has no problem to join and generate new keys, without having to be reprogrammed.

However, in our particular case, there is a mobile node that can transmit to several gateways. Their relative distances to the vehicle change, and therefore their RSSI with respect to the end devices. This variation of the RSSI forces the restart of the procedure for joining the network, and changes the session keys. Thus, nodes are unrecognizable if more than one packet forwarder (gateway) is covering the vehicle during its journey. Thus, multicasting is not allowed in OTAA mode, and then it is not useful for the proposed use case.

#### 3.3.2. Activation by Personalization

**Figure 5:**
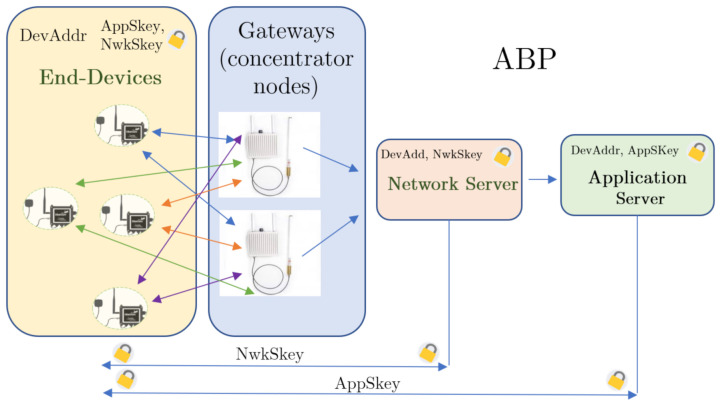
Activation By Personalization (ABP) scheme.

In ABP activation mode (Activation By Personalization), the network server is not required to accept a request for a specific end-device so that they can exchange messages, i.e., a join-request is not necessary. With respect to the OTAA mode, the structure changes, since in this case the AppKey does not have to be stored by the motes (Figure 5). The configuration of the end-device is done by a physical address (DevAddr), which must be known to the network server. In addition, the application and network session keys (AppSkey and NwkSkey) are pre-programmed in each end-device, that is to say they are now statically generated. The network server only needs to know the device address and the network session key, while the application server stores the application session key and the end-device address.

Thus, when the end-device wants to communicate, it does so using the session keys, without having to perform a joining procedure first. Therefore, this method allows direct communication between the devices and the servers, through all the gateways, but the keys may be compromised, as any corrupted node would expose the keys of the whole network. To avoid the data packet replay attacks, it is normal to use a mechanism that changes the session keys every time the nodes are reset [71].

Then, ABP mode allows any end-device to transmit its data directly to the network server, which is encrypted and signed. All LoRaWAN devices need to encrypt their payloads and headers using an algorithm based on the AES (Advanced Encryption Standard), using 128-bit keys. The LoRaWAN protocol offers two layers of security, managing to protect the information from end to end:

- In the network layer, the message integrity is established by the MIC, making use of the NwkSkey in order to encrypt the payload from end-devices to the network server.
- In the application layer, the payload is encrypted using the AppSkey so that the payload is encrypted from end-devices to the application server.

All dataframes contain a sequence number that uniquely identifies them within each gateway. This prevents several gateways from receiving the same packet from a given sensor node, since it cannot acknowledge transmission simultaneously with different keys. This means that OTAA does not allow multicasting when the gateways are working in local mode, as it was verified in several tests previous to the experiments. Our application requires ensuring that, at least, two gateways can simultaneously receive data from the same sensor node, so that it is possible to compare the number of packets received in different sites. This way, ABP mode has been chosen. Thus, comparing the packets received by the gateway onboard the vehicle with the external ones, it is concluded which areas are susceptible to not receiving messages. This allows us to establish location strategies for gateways in Málaga, discarding the less efficient locations.

### 3.4. Operating Modes of the System

One important goal of this paper is to compare LoRa configurations for establishing LoRa message communication strategies in an urban environment, as well as the site location for static gateways. In order to make these comparisons, an external database (hosted on a web server) has been created, in which a series of tables have been established, associated with each of the gateways. To transfer data to it, a portable program has been developed to allow downloading the data from the different gateways, in real-time, into a database, being able to operate in two modes (called offline and online). The concentrator nodes have to be continuously fed through a PoE (Power Over Ethernet) during the course of the experiments to collect the acquired data. They do not need to be connected to the Internet, because each gateway has memory enough to take frames for two months or 200 MB, with no danger of memory saturation. Actually, the possibility of synchronizing all the gateway readings from certain times of the day has been established, in order to extend the filing of its internal memory over time. For this, two operating modes are presented below:

- Offline mode. The information is taken in plain text files that are interpreted and grouped in files of type .CSV, for further treatment. The data are stored in the laptop computer (control station embarked on the car) through a developed portable GUI, from which they can be uploaded to the external database as soon as the Internet is available. The advantage is that it is not necessary to have an access point to the Internet in order to collect information, since the analysis and processing of the data will be done when the period of experimentation is over. This mode is therefore suitable for networks whose deployment has a limited duration (several days or weeks) and in which the processing of the data does not need to be done in real-time. This is possible because the packet forwarders have an MQTT server installed onto them, which subscribes to the topics published by the sensor nodes.
- Online mode. The principle is the same, but now each gateway is connected to an Internet access point (through a 4G SIM card or a router), so that, in real-time, they dump received data into the external database and, in turn, into a web server with Google Maps services. In this way, it is possible to view the data collected in the frames, with their geo-location, as shown in the experiments.

Therefore, there is the possibility of receiving what is happening in the vehicle at locations far away from the vehicle, without the need to use the Internet. This could be interesting in some cases, when it is preferred to avoid the risks of hacking via the Internet (system acting as LoRa Local Area Networks), or simply it is not necessary to obtain the information from the network, until some time has passed (which is limited by the internal memory of the gateways). Then, it is not necessary to have a gateway in the vehicle, but only the sensors, and that these transmit by radio to the gateways located in the buildings. However, as explained above, the gateway onboard the vehicle allows for assessing static gateway sites in the urban environment of the experiments.

### 3.5. Mobile Node

The mobile node has been developed to test the functionality of LoRa in different areas of the city, being able to evaluate how obstacles and interferences in the city influence transmissions. In order to supervise the experiments, the control centre has been mounted in the vehicle itself. It consists of a laptop connected via RJ45 cable to the gateway, whose antenna is attached to the vehicle’s roof rack.

As mentioned earlier, the goal is to test LoRa technology in an urban environment. A previous work, providing a solution based on Zigbee [68] is used as a baseline to compare results of the proposed system, as well as to verify any improvements in this scenario.

The purpose is to measure the environmental parameters of the city of Málaga. In the previous work, a receiver device (Meshlium) was mounted on the vehicle, so that a mobile gateway was available. The sensor nodes were located on the roof of the car, so that a coverage failure was unlikely to occur (despite the short range of Zigbee), given the proximity between the transmitting and receiving antennas. However, the aim of that project was to create an Urban Information System (UIS), with a local database, from a single gateway. A SIM card was inserted in the gateway on board the vehicle, which managed the data directly. Given the short range of Zigbee (about 100 m), the mobile node was intended to provide additional information with respect to a static network, both carrying sensor nodes or acting as a mobile sink. The integration of a mobile node made the sensor network a hybrid one.

In the proposed system, based on LoRa, the concept has changed: the goal of the mobile node is to check coverage to determine the best configuration of sites for static gateways, instead of providing coverage by means of a mobile sink. Therefore, the resulting system has transitioned from a hybrid wireless sensor network (H-WSN) to a hybrid network of gateways (static and mobile). In fact, it would not be necessary to use the in-vehicle gateway if the goal is to pick up packets from existing in-vehicle sensors, while it is being driven around the streets. However, there are two reasons for using the mobile concentrator node:

- To compare the data packets received by the mobile gateway to those taken by the external static gateway network. In this way, it is possible to establish which zones of the city are white, which means that they are not covered by the mobile node, nor by the static concentrator nodes located at high points around the city.
- To listen to data packets from sensors embarked on the vehicle to compare the range and to count the dataframes acquired by the LoRa gateway with respect to the Zigbee gateway used in [68], since both of them have been driven along the same route. In addition, this allows us to analyze the behavior of a LoRa gateway in motion, at normal urban traffic speeds.

The transmitter modules on board the vehicle create frames of different sizes, always within the size range allowed by LoRa technology (from 51 to 222 bytes), depending on the Spread Factor used (from 12 to 7, respectively). However, the idea is to gather all the data taken by all the end-devices as if they were sent by a single modular node: the mobile transmitter node, that is to say, the vehicle. In this way, the size and power limitation of LoRa is solved, being able to group different magnitudes taken by different nodes in the same packet, which will have associated the geo-location taken by one of the GPS antennas present in the vehicle.

**Figure 6:**
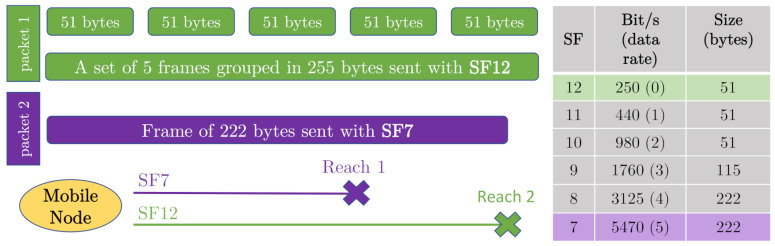
Small frames grouped to obtain more reach.

As it can be seen in the diagram in Figure 6, virtual packets have been obtained by merging small real packets. Small packets allow for using a higher spread factor, which improves range. Besides, greater redundancy is achieved in the delivery of a packet consisting of several measurements taken by different sensor probes. This way, more information can be sent, farther away, and with a greater guarantee of reception, if the devices, on board the vehicle, send small packets with the maximum spread factor (SF12), instead of sending medium or large packets with the minimum spread factor (SF7).

The union of small data frames in a big message is done by software, and it is managed in the database, establishing a time interval for the acceptance of different packets in the same frame. Therefore, a temperature read by the probe of module A, can be grouped with the concentration of $CO_{2}$ read by another module B and, at the same time, with the geo-location taken by the GPS antenna of another modular end-device C. In the user application, a message will be shown constituted by the union of the packets coming from different transmitter node units. This can be done because all the transmitters are located in the same physical place (relatively), since they move together with the vehicle, recording the information of the city.

## 4. Experiments

A series of experiments has been carried out to test the behaviour of LoRa technology in an urban environment. The experiments comprised a series of tests, although all the data shown in this section belong to the same experiment. It should be noticed also that before actual experiments, a set of tests were carried out to check different configurations of the system, like the number and placement of sensor nodes, data rates, packet lengths, etc. These tests were performed on the Campus, where a limited version of the setup for the experiments in the city was re-created. A static gateway was installed, and several configuration tests were carried out, to determine the most promising configuration. The final configuration is summarized in Table 1. Higher SF were chosen to obtain a better penetration, since the goal was an urban scenario. This higher SF involves reducing the white areas, within each zone of interest, seen by each static gateway, since packets are stronger against interferences of the band and obstacles. The mobile node was configured with a set of sensor nodes (see Table 1) as well as a concentrator node. The vehicle performed a route along the city, similar to that of [68]. The route took 90 min of actual time to be completed, substracting stops, and it was performed during peak times. Several static gateways have been deployed in different locations of the city of Málaga.

**Figure 7:**
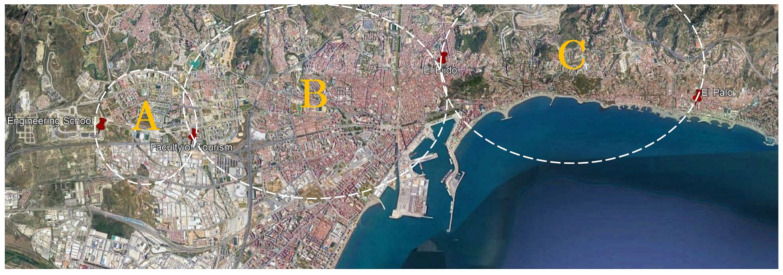
Location of the static gateways.

Three circular areas can be established, each of which has as its diameter the distance between two consecutive gateways along the route (Figure 7). The vehicle is driven from west to east of the city of Malaga, emitting and receiving packets in each area.

The mobile gateway operates in multicasting with the gateways of each area (A, B and C), thanks to the ABP activation mode. All the gateways have their own MQTT server running, independent from the other ones, which allows us to operate without an Internet connection to store the data. The plain text files generated in its internal memories save all the information below the topic lora/+/up, which means that all the uplink packets sent by any LoRa end-device (+indicates any DevEUI) is going to be recorded (in JSON format). If the system is operated in the offline mode (explained in Section 3.4), these files are read after the experiments. In the online mode, it is possible to dump and monitor all the information saved in the plain text file, since they will be synchronized with an external database hosted on a web server, linked thanks to the developed application (lorApp).

**Figure 8:**
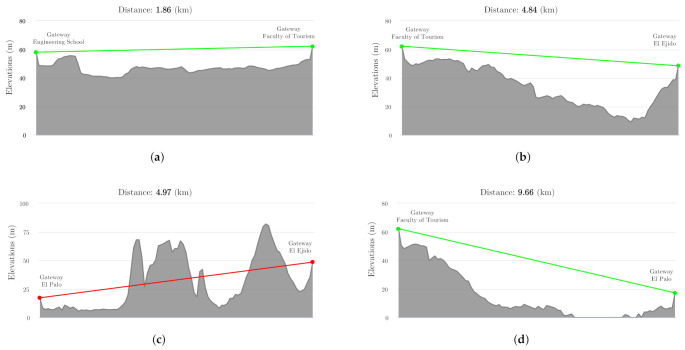
Lines of sight between receiving antennas located in the path of the mobile node. (**a**) Line of sight between School of Engineering and Faculty of Tourism (zone A). (**b**) Line of sight between Faculty of Tourism and El Ejido (zone B). (**c**) Line of sight between El Ejido and El Palo (zone C). (**d**) Line of sight between Faculty of Tourism and El Palo (9.66 km).

Figure 8 shows the ground profile between two consecutive gateways to see the different lines of sight along the itinerary of the vehicle. As can be seen, the view between the receiving antennas is not always good, which is why several ones have been used along the vehicle’s route. This allows the evaluation of each zone of the city along the route, comparing the packets received by the static gateways with those collected from the vehicle. It has to be noticed whether or not the buildings around each of the gateways affect the suitability of their location. For instance, in Figure 8c, it can be seen that the line of sight between the gateways located further east (zone C) is completely null due to the opaque profile of the terrain between them.

This means that the area between these static gateways is not completely swept from their locations and multicasting does not exist between them but it does with the mobile concentrator when it goes through the zone covered by each of them. In addition, this area is characterized by a high density of buildings, of equal height, which affects the signal’s penetration capability. Thus, inside area C (4.97 km in diameter), most of the transmitted packets from the mobile sensors have been lost by both of the static gateways in range, but received on the mobile gateway, which verifies the influence of the line of sight on the LoRa transmissions. In addition, these two static gateways are located in denser urban areas than those covered by the first two gateways (located further west), which poses a greater challenge for penetration, even for those nodes that were programmed with a high spread factor (11 or 12). Although the terrain profile had previously been studied to establish the static gateway positions, the intention was to verify experimentally that static gateways located in these areas would count fewer packets, and their radius of action would be considerably smaller.

Finally, the use of the gateway located further east (zone C) in the city was discarded as it did not contribute enough to the collection of packets, but it was useful in verifying the limited usefulness of LoRaWAN in areas of great terrain variability and many obstacles when good visibility is not possible. Reducing the percentage of transmission obstacles in this area (C) would require a much higher elevation of the static gateway (for example, by locating it on a nearby mountain), but such a structure was not available in the zone.

**Figure 9:**
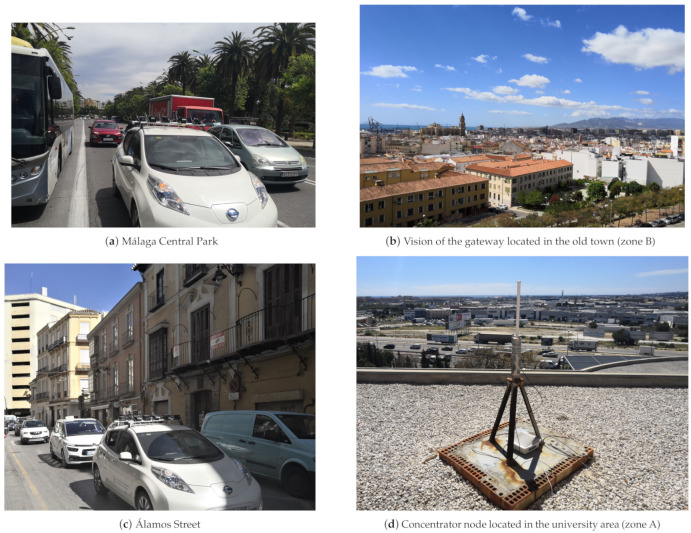
Sensor carrier vehicle acting as transmitter/concentrator mobile node, and view of two external gateways.

In Figure 9, the vehicle is shown carrying the sensors as it passes through the area B, which has the highest density of obstacles (buildings, trees, trucks, etc.) along the way (Figure 9b). This figure also shows the excellent field of view of both static gateway antennas which sweep zone B, from an elevated level (Figure 9b,d). Therefore, in spite of the fact that the environment presents a great density of obstacles, the locations of the static gateways allow us to obtain great coverage.

Each sensor node has a unique identifier and an address known by each concentrator node (packet forwarder), so it is possible to know which sensor node is emitting more or fewer packets, and to count how many dataframes are read by each gateway from each sensor node. This allows for assessing the quality of the chosen locations for static gateways around the itinerary. In addition, the configuration is simple and the network is quite flexible, since it allows us to add more transmitter nodes both in the city (static network) and in the vehicle (mobile node), being able to create a more extensive hybrid sensor network depending on the needs.

Table 2 and Table 3 show the number of packets that have passed through the static gateways that provide coverage to zone B. Table 4 shows the same for the mobile gateway embarked in the vehicle, which corresponds to the maximum number of packets received in the network, given the proximity between this packet forwarder and the eight end-devices embarked.

These tables include the list of the transmitter nodes on board the vehicle, listed by DevEUI and Network Address. It must be noticed that the four gas sensor nodes share two identifiers, due to their slow dynamics. In fact, every type of sensor node has its own dynamics, as it can be observed through the different number of transmitted packets. Therefore, the last two DevEUIs of the list, associated with gas sensors, whose dynamics is very slow (they require several minutes to measure and create a single packet), have a much lower value than the rest of the nodes (which measures, for example, temperature, pressure or GPS data) in the packet count. Furthermore, in Table 4 it can be seen that the number of lost packets was very low. Of the 995 LoRa packets emitted by all the transmitting nodes, only 47 were lost (4.7% of the total). This loss is associated with packet collisions. This is obtained from the difference between uplink (UL) and downlink (DL) packets, since when an end-device emits a packet, it must receive the corresponding acknowledgement (ACK message) from the gateway located in its coverage radius. Thus, the maximum number of received packets is given in the mobile gateway (948), and of these, each static gateway located around the city, will receive a percentage.

In addition, the tables inform whether the packets have arrived in the first or second reception window, opened by the class A devices. The recorded RSSI and SNR values have been included, which are logically worse the farther the gateways are from the node-carrying vehicle. With respect to the column labeled as Seq Num, it only indicates the historical number of packets that each specific packet forwarder has received from the corresponding node, in the total of experiments performed.

**Figure 10:**
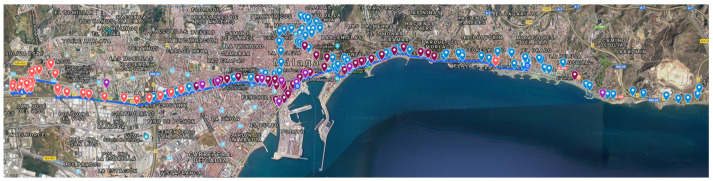
Data received by the different gateways.

The Google My Maps server has been used to visualize the data recorded by each of the gateways (packet forwarders), being able to measure the transmission distances achieved, as well as to geo-locate the measurements taken by different sensors. For that purpose, a specific color has been assigned to each concentrator node, so it is easy to check the coverage of each receiving antenna outside the vehicle (Figure 10). Many points appear superimposed on the map, due to multicasting.

**Figure 11:**
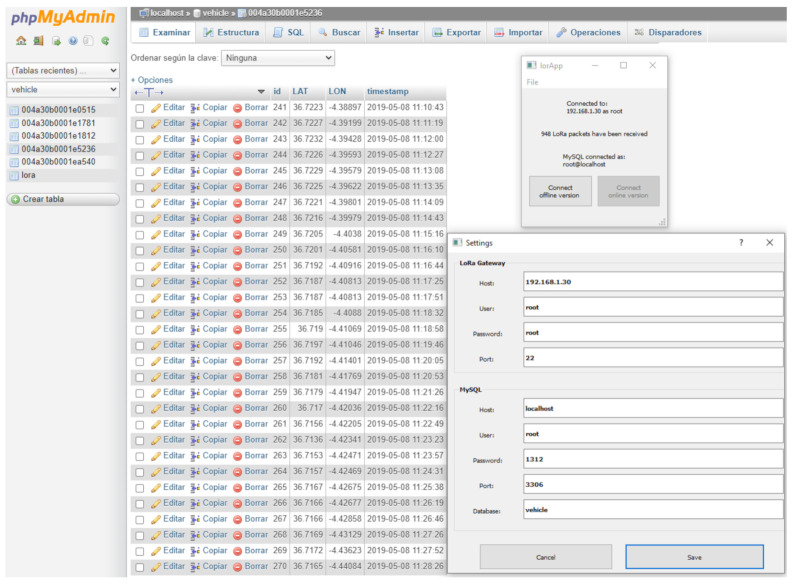
LoRa packets received at the network server during the experiment.

The program developed (lorApp) is capable of processing and dumping the data packets in a local or external database (Figure 11), as well as associating it with the Google My Maps web server. This allows to count and visualize the number of packets received by each concentrator node. Each external gateway covers areas of the city not seen by other gateways, so that they complement each other.

**Figure 12:**
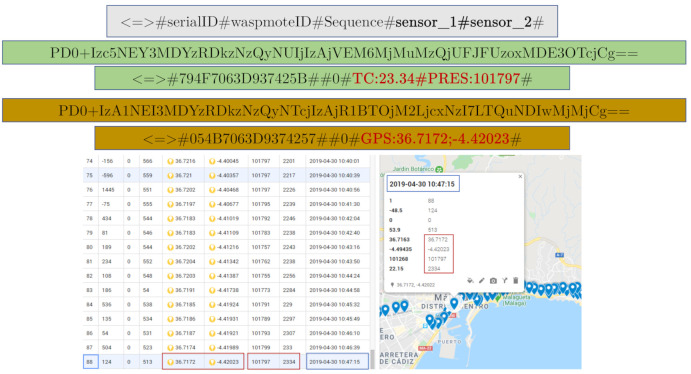
Dataframes structure.

Figure 12 shows the dataframe’s structure used and how two packets issued by two different sensor nodes (both onboard the vehicle) have been grouped. In the case shown, one of them measures and transmits (via LoRa) the temperature and pressure, and the other one only the GPS position of the vehicle. It should be noted that the payloads are encoded in base 64. In the application server, this information has been combined, and a larger packet can be obtained at a greater distance than the LoRa technology itself allows. For that, several pre-programmed Structured Query Language (SQL) sentences have been used to join tiny packets emitted by different end-devices. Thus, the LoRa Network Server can receive more information from positions farther away from the vehicle due to the use of an SF12 on all nodes, except for one that has been configured with SF8 (higher data rate and lower robustness) to transmit more information simultaneously (in the same structure of data) and to study the difference between two extreme spread factors. Then, this last node has successfully transmitted fewer packets due to interference and obstacles. The small packets are related to each other thanks to the timestamp implicit in each one of them. Thus, it is possible to assign an updated GPS position to the resulting packet, while the vehicle is moving on.

**Figure 13:**
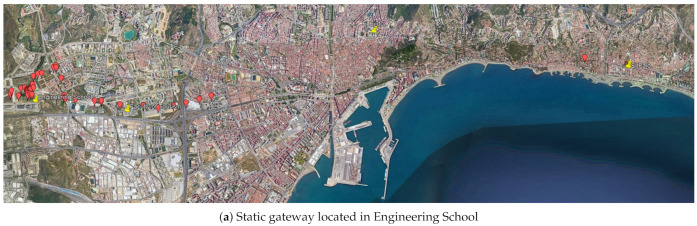

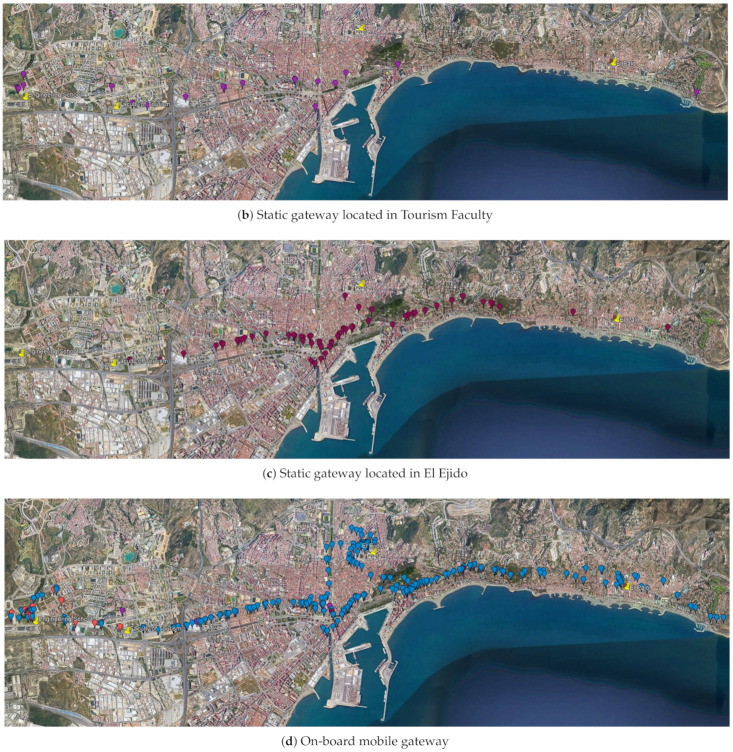
Receiving LoRa packets at the gateways.

Table 5 shows the number of packets received by the static gateways, together with the packets received by the gateway at the mobile node. Figure 13 shows packets captured by all the gateways. A total of 554 LoRa packets were received at the static gateways from the total number of packets emitted from the mobile node, which represents 58.43%.

Finally, it has been possible to get packets from the mobile node at a maximum distance of 7 km, through the urban environment. Thanks to the good line of sight between the vehicle and some of the different static gateways, during the trip, some packets have achieved such great ranges despite being an urban environment. It should be noted that some packets traveled over the sea (altitude 0 m), which suggests possible locations of transmitter nodes near the port since they could be seen by the most successful gateway, which has turned out to be the one located on El Ejido’s university campus.

**Figure 14:**
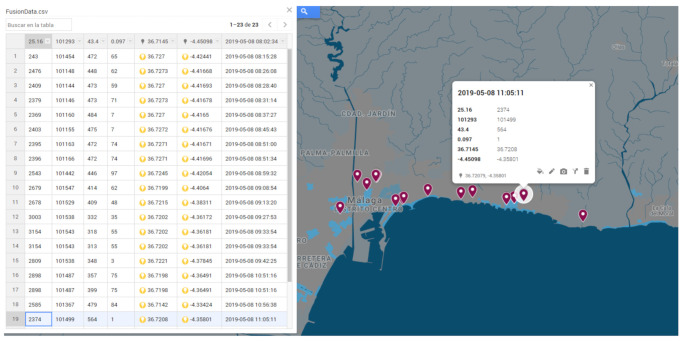
Captured data from three different nodes and their visualization.

Comparing Figure 13d with Figure 13a–c, white areas for every gateway can be identified. It can be seen that the static gateway located at El Ejido leads to smaller white areas in the city, which in turn could be further reduced using the byte format instead of the ASCII format to send the same information. In order to reproduce the length of the packets sent via Zigbee in [68], it was decided not to use the byte format for these experiments. However, it can be expected that configuring all the four gateways with byte format will reduce the white areas for the whole city. Another future line of work is the extension to other areas in the city, with different terrain, including simulations of radio coverage prior to experimental measurements. Table 6 shows some packets obtained by the hybrid network of concentrator nodes, from three different end-devices (two of them programmed with SF12 and the other with SF8), whose data has been combined and monitored (Figure 14). The sampling time is limited to the slowest dynamics of the sensors (in this case, ozone).

However, much more up-to-date data is available when dealing with fast dynamic sensors. Here, a difference can be made with respect to the experiments carried out with Zigbee, in the sense that more information is obtained with the same number of nodes, and from farther away. For instance, the number of packets received in the static gateways were 554 packets in this experiment. However, in the Zigbee series of experiments in [68], the number of packets only reached 136 for a similar route that took a similar time to complete.

## 5. Conclusions

A hybrid network of long-range concentrator nodes and wireless sensors has been presented. The system architecture includes several static gateways and a modular mobile node. This mobile node is set up on an electric vehicle, and is capable of using different kinds of sensors according to mission needs. In addition, a LoRa gateway has been included, allowing the mobile node to act not only as a transmitter but also as a sink of urban information. This way, the mobile node receives all the dataframes sent by the sensor nodes onboard, allowing for a comparison with those received in the different static gateways. Besides, the mobile node adds flexibility and range to the sensor network. The architecture is flexible enough to change the number and type of sensors easily and also to modify the locations of the external concentrator nodes so that the service provided is better as possible. Experiments have been performed with a different set of sensors, showing no compatibility problems. The modular design of the mobile node allowed an easy reconfiguration.

The system has been deployed and tested in a mid-size city (Málaga, Spain, with around 500,000 inhabitants). A static network of concentrator nodes has been deployed at different points in the city in order to cover as much area as possible. The vehicle with the mobile node has followed a route across the city during a series of experiments. A previous work testing a different sensor network has been used as a baseline to compare results. Thus, the route during the experiments in this work follows a similar route to that in [68].

Activation By Personalization mode has been used to communicate data between end-devices and gateways, the choice of which is justified for allowing multicasting. This way, as the mobile concentrator node moves through the city, its radius of action interacts with the radius of action of another static concentrator node, both of which must be able to listen to the same packet emitted by the same sensor node at a specific moment (timestamp). This allows us to compare the real coverage of each static gateway. With this methodology, the experiments show that the city of Málaga can obtain service through a single gateway located in the El Ejido Government Pavilion building. This gateway provides a coverage radius wide enough to deploy many applications of the Internet of Things in this city, thanks to the excellent line of sight, but also to the configuration established in the different sensor nodes. With high spreading factors (SF11 o SF12) and small payloads (of the order of 50 bytes), the communication tends to be more robust against interference and obstacles, and to achieve a longer range. The experiments have shown how this location works well for exchanging information with objects, vehicles, or people on the roads in the old town and beyond. In particular, for this gateway, almost 35% of LoRa packets issued from the vehicle have been collected, even higher than the other two static gateways combined.

Considering the location of the static gateways, it can be noticed that dataframes were sent, and received by at least one static gateway, along with a distance of 12 km. Since there were three active static gateways (the fourth eastwards being discarded due to being too enclosed by surrounding buildings), it can be noticed that the actual range surpassed 5 km, which is the expected range of LoRa in an urban environment, according to [19].

When compared with the previous application in [68], tested in the same area, and with a similar route, the system based in LoRa provides a higher number of packets received: 554 against 136. In the mentioned work, coverage for the mobile node was provided using 3G. Synchronization with the external database took advantage of a commercially optimized network. In the case of the LoRa system, coverage of the mobile node (apart from that of the embarked gateway) was provided by the static gateways. Despite that fact, the number of packets received was higher, as mentioned above.

As a conclusion, using a mobile node together with ABP activation mode allows for multicasting without the need for an internet connection. This combination makes it possible to test coverage for several static nodes around an area of interest. Static gateways should be placed in sites as high as possible, but also looking for clear areas in order to avoid reflections (as shown in the case of the static gateway at El Palo, with an appropriate height but surrounded by buildings). The use of high spreading factors (meaning longer times of flight for packets, which in turn implies a higher probability of reception) and small payloads promises a better behavior against interferences and obstacles, although with higher energy consumption, which can be acceptable in a city application.

The flexibility of the system makes it easy to be adapted to other use-cases, like search and rescue missions, where its capability to work independently from commercial networks (i.e., cellular networks) can be valuable. Future lines of work consider an extension of the proposed system to emergency applications, allowing collaborative efforts between different agents integrated into the mission, such as robots, drones, dogs and humans, which can have sensors attached. The same set of parameters presented in Table 1 can be a starting point for this kind of application, since having a direct sight is difficult to predict. Codification in byte format, instead of ASCII, might be preferable. This option reduces the size of the data packets at the cost of a harder decodification. However, smaller packets promise more robustness against interferences. This alternative was checked in the configuration tests previous to the actual experiments. However, for the sake of comparison with [68], an ASCII codification was preferred.

## Figures and Tables

**Table 1:** LoRa end-devices embarked on the vehicle.

End-Device	Units	SF	Sent Bytes per Unit	Measurements	Other Parameters of Interest
Smart Environment PRO	2	12	50	${CO}_{2}$, ${NO}_{2}$, $O_{3}$, CO	RSSI, SNR, seqn, timestamp
Smart Environment PRO	2	9	88	${CO}_{2}$, ${NO}_{2}$, $O_{3}$, CO		
Ambient Control	3	12	50	Temperature, pressure. humidity and/or GPS	
Radiation Control	1	12	50	GPS	

**Table 2:** LoRa packets received by the static gateway located in faculty of tourism.

Net Addr	Dev EUI	Class	Joined	Seq Num	Up	Down	1st	2st	RSSI min	max	avg	SNR max	min	avg
00:00:10:01	00-4a-30-b0-00-1e-18-12	A	2019-04-29T16:56:47z	801	16	16	8	8	−114	−52	−91	−15.8	10	2.8
00:00:10:02	00-4a-30-b0-00-1e-a5-40	A	2019-04-29T16:57:34z	709	26	20	6	18	−119	−65	−97	−16.2	9.5	6.3
00:00:10:03	00-4a-30-b0-00-1e-17-81	A	2019-04-29T16:57:45z	2152	30	29	11	18	−121	−57	−88	−14.8	11.7	7.7
00:00:10:04	00-4a-30-b0-00-1e-17-83	A	2019-04-29T16:57:55z	659	12	10	4	6	−121	−59	−95	−17	13.3	5.8
00:00:10:05	00-4a-30-b0-00-1e-8a-09	A	2019-04-29T16:58:11z	82	2	2	1	1	−115	−56	−98	−8.6	13.1	6.2
00:00:10:06	00-4a-30-b0-00-1e-52-36	A	2019-04-29T16:58:26z	325	5	3	2	1	−122	−58	−88	−18	11.5	7.5

**Table 3:** LoRa packets received by the static gateway located in El Ejido (old town).

Net Addr	Dev EUI	Class	Joined	Seq Num	Up	Down	1st	2st	RSSI min	max	avg	SNR max	min	avg
00:00:10:01	00-4a-30-b0-00-1e-18-12	A	2019-04-29T16:56:47z	705	64	61	20	41	−121	−55	−93	−17.8	12	3.7
00:00:10:02	00-4a-30-b0-00-1e-a5-40	A	2019-04-29T16:57:34z	722	80	77	35	42	−120	−67	−95	−18.2	11	4.6
00:00:10:03	00-4a-30-b0-00-1e-17-81	A	2019-04-29T16:57:45z	2486	103	101	52	49	−121	−58	−92	−16.2	12.2	7.8
00:00:10:04	00-4a-30-b0-00-1e-17-83	A	2019-04-29T16:57:55z	742	58	58	25	33	−121	−58	−99	−17	13.2	5.9
00:00:10:05	00-4a-30-b0-00-1e-8a-09	A	2019-04-29T16:58:11z	91	18	14	10	4	−119	−55	−97	−8.5	13.5	6.8
00:00:10:06	00-4a-30-b0-00-1e-52-36	A	2019-04-29T16:58:26z	285	14	9	3	6	−123	−58	−89	−17	12	6.5

**Table 4:** LoRa packets received by the mobile gateway.

Net Addr	Dev EUI	Class	Joined	Seq Num	Up	Down	1st	2st	RSSI min	max	avg	SNR max	min	avg
00:00:10:01	00-4a-30-b0-00-1e-18-12	A	2019-04-29T16:56:47z	754	141	134	125	4	−75	−21	−39	−1.7	6.5	4.1
00:00:10:02	00-4a-30-b0-00-1e-a5-40	A	2019-04-29T16:57:34z	779	365	359	349	8	−78	−26	−43	−1.4	7	3.6
00:00:10:03	00-4a-30-b0-00-1e-17-81	A	2019-04-29T16:57:45z	2777	229	215	101	105	−81	−30	−39	−2.5	5.5	4.1
00:00:10:04	00-4a-30-b0-00-1e-17-83	A	2019-04-29T16:57:55z	3026	195	187	80	97	−79	−25	−37	2.3	4.1	3.0
00:00:10:05	00-4a-30-b0-00-1e-8a-09	A	2019-04-29T16:58:11z	156	39	32	32	0	−87	−22	−29	1.6	5.3	2.7
00:00:10:06	00-4a-30-b0-00-1e-52-36	A	2019-04-29T16:58:26z	312	26	21	15	6	−80	−24	−31	3.5	6.1	4.1

**Table 5:** LoRa packets received at each gateway.

Gateway	Vehicle	Engineering School	Faculty of Tourism	El Ejido (Old Town)
Packets counted	948	144	80	330
LoRa packets (%)	100	15.19	8.44	34.81

**Table 6:** Combined data from three nodes onboard the vehicle.

T (℃)	p (Pa)	RH (%)	O${}_{3}$ (ppm)	Latitude (${}^{\circ }$)	Longitude (${}^{\circ }$)	Time
24.3	101,454	47.2	0.065	36.727	−4.42441	08:15:28
24.76	101,148	44.8	0.062	36.7273	−4.41668	08:26:08
24.09	101,144	47.3	0.059	36.727	−4.41693	08:28:40
23.79	101,146	47.3	0.071	36.7273	−4.41678	08:31:14
23.69	101,160	48.4	0.07	36.727	−4.4165	08:37:27
24.03	101,155	47.5	0.07	36.7272	−4.41676	08:45:43
23.95	101,163	47.2	0.074	36.7271	−4.41671	08:51:00
23.96	101,166	47.2	0.074	36.7271	−4.41696	08:51:34
25.43	101,442	44.6	0.097	36.7245	−4.42054	08:59:32
26.79	101,547	41.4	0.062	36.7199	−4.4064	09:08:54
26.78	101,529	40.9	0.048	36.7215	−4.38311	09:13:20
30.03	101,538	33.2	0.035	36.7202	−4.36172	09:27:53
31.54	101,543	31.8	0.055	36.7202	−4.36181	09:33:54
31.54	101,543	31.3	0.055	36.7202	−4.36181	09:33:54
28.09	101,538	34.8	0.003	36.7221	−4.37845	09:42:25
28.98	101,487	35.7	0.075	36.7198	−4.36491	10:51:16
28.98	101,487	39.9	0.075	36.7198	−4.36491	10:51:16
25.85	101,367	47.9	0.084	36.7142	−4.33424	10:56:38
23.74	101,499	56.4	0.1	36.7208	−4.35801	11:05:11
25.98	101,523	52.4	0.128	36.7225	−4.39622	11:13:35
26.03	101,496	50.3	0.09	36.7201	−4.40581	11:16:10
26.08	101,506	50.3	0.09	36.7192	−4.40916	11:16:44
29.01	101,402	41.4	0.07	36.7169	−4.43129	11:27:26

## Data Availability

The data presented in this study are available on request from the corresponding author.
